# The Influence of Edible Oils’ Composition on the Properties of Beeswax-Based Oleogels

**DOI:** 10.3390/gels8010048

**Published:** 2022-01-09

**Authors:** Yuliya Frolova, Varuzhan Sarkisyan, Roman Sobolev, Mariia Makarenko, Michael Semin, Alla Kochetkova

**Affiliations:** 1 Laboratory of Food Biotechnology and Foods for Special Dietary Uses, Federal State Budgetary Scientific Institution Federal Research Center of Nutrition, Biotechnology and Food Safety, 109240 Moscow, Russia; sarkisyan.varuzhan@gmail.com (V.S.); sobolevrv@bk.ru (R.S.); vip-planckton@yandex.ru (M.S.); kochetkova@ion.ru (A.K.); 2 Laboratory of Food Chemistry, Federal State Budgetary Scientific Institution Federal Research Center of Nutrition, Biotechnology and Food Safety, 109240 Moscow, Russia; dragon.soul1992@ya.ru

**Keywords:** oleogel, beeswax, oil composition, physicochemical properties, correlational analysis

## Abstract

This study aimed to find relationships between the properties of beeswax-based oleogels and the type of oil used. The influence of linseed, sunflower, olive, and fish oils was studied. For these oils, the fatty acid composition, the content of total polar components, and the iodine value were characterized. Textural and thermodynamic properties were determined for oleogels, the oil-binding capacity was estimated, and the morphology of crystals was studied. The concentration of beeswax in all oleogels was 6.0% *w/w*. It was shown that the type of oil has a significant influence on all characteristics of the oleogels. The use of different oils at the same technological treatment leads to the formation of crystals of diverse morphology—from platelets to spherulites. At the same time, it was revealed that some characteristics of oils have a varying contribution to the properties of oleogels. The content of total polar materials in oils is associated with a decrease in strength parameters (yield value and elastic modulus) and the oil-binding capacity of oleogels. In its turn, the iodine value of oils has a close positive correlation with the melting and crystallization temperatures of oleogels. The results obtained in this article indicate that the properties of beeswax-based oleogels can be directed by changing the oil composition.

## 1. Introduction

The growing interest in the use of oleogels as an alternative method of structuring liquid edible oils is related to the need for reducing the consumption of saturated and *trans*-fatty acids from food products [1,2]. Currently, different research groups have produced oleogels containing liquid edible oils: sunflower oil [3], linseed oil [4], olive oil [5], fish oil [6], and others [7,8,9]. Edible oils differ from each other primarily by their fatty acid composition. The most common fatty acids of liquid edible vegetable oils are long-chain unsaturated oleic (C18:1), linoleic (C18:2), linolenic (C18:3), and very-long-chain fatty acids eicosapentaenoic (C20:5) and docosahexaenoic (C22:6), which are components of animal oils [10]. The use of structuring techniques for liquid edible oils makes it possible to obtain semi-solid systems (oleogels), which retain a solid consistency without adding solid fats, which are triglycerides with saturated or *trans*-isomeric acids [11]. Waxes, particularly beeswax, are among the most common structure-forming agents for the production of oleogels. Beeswax is a complex mixture that includes various components. Among them are four major classes: hydrocarbons, esters, free fatty acids, and alcohols [12]. The ability of waxes to structurize oils depends significantly on the wax composition and the rate of cooling [13]. Currently, various teams of authors have conducted studies on the ability of beeswax to structure only certain liquid vegetable oils. The exact composition of the used beeswax is not always indicated, which makes it difficult to compare the properties of the obtained gels between individual researchers. The main range of investigated concentrations of the structure-forming agent in oleogels is from 0.5% *w/w* to 10.0% *w/w*, and the critical concentration of gel formation for beeswax for the majority of oils is 6.0% *w/w* [14,15]. At the same time, the influence of oil type on the structuring process of oils by beeswax has been insufficiently studied. Analysis of existing works indicates the specificity of interaction of the structuring agent with oils. For example, ethylcellulose-based oleogels form stronger gels in linseed oil compared to soybean and canola oil oleogels [16]; however, the addition of adipic acid significantly improves the properties of ethylcellulose and soybean oil oleogels [17]. In its turn, the combination of γ-oryzanol with β-sitosterol has been shown to form stronger gels in sunflower oil [18]. These differences may be related to the mechanism of gel formation and the interaction of the structure-forming agent with the oil [13]. The fatty acid composition of oils, as the main distinguishing feature of all edible oils, should influence the properties of oleogels in the first place. Depending on the content of saturated fatty acids, oils have different crystallization and melting temperatures, which can affect the crystallization processes in oleogels. In addition, the polar materials of nonlipid nature (which are part of oils) can have their role [19,20]. These materials are diverse, but what they have in common are their low molecular weight and high polarity. The importance of minor substances has been shown, particularly for gels based on ethylcellulose in olive oil [21]. A variety of factors can influence different characteristics of oleogels, which are unrelated to each other. At present, there is no systematic understanding of the direction of the impact of the individual factors of the oils’ composition on the properties of beeswax-based oleogels. At the same time, obtaining oleogels with the specified for certain types of food products requires an understanding of the role of all the components in the composition of the product on the properties of the oleogel.

Thus, the determination of the relationships between the composition and properties of oils and the properties of the obtained oleogels based on beeswax would allow for their tailored design. Given that, this study aimed to investigate the gel-forming ability of beeswax in the composition of oleogels based on vegetable and animal oils with a high content of unsaturated fatty acids.

## 2. Results and Discussion

### 2.1. Edible Oils Analysis

The fatty acid composition of the studied oils was characterized (Table 1). The values obtained were within the acceptable range for a particular type of oil [10].

As can be seen from Table 1, the lowest content of unsaturated fatty acids (UFA), as well as the highest content of saturated fatty acids (SFA), is shown for fish oil. The highest content of polyunsaturated fatty acids (PUFA) was noted for sunflower and linseed oils. Olive oil contains the highest amount of monounsaturated fatty acids (MUFA). The presence of very long-chain fatty acids (C ≥ 20) is typical exclusively for fish oil. Among them, there are polyunsaturated fatty acids with five or more unsaturated bonds, particularly eicosapentaenoic (C20:5), docosapentaenoic (C22:5), and docosahexaenoic (C22:6) acids. These acids are absent in the studied vegetable oils, in which PUFAs are represented by linoleic (C18:2) and linolenic (C18:3) acids.

Therefore, direct assessment of the influence of oil fatty acid composition on the properties of oleogels is limited due to the almost complete absence of very-long-chain unsaturated fatty acids (C > 20) in vegetable oils. In this connection, the iodine value (Table 1) was calculated for each sample as an integral index of the degree of unsaturation of the oils based on the obtained data. The highest value of cIV was observed for FO (164.18 g) and LO (160.38 g) samples. The lowest value of cIV is characterized by olive oil (85.33 g). In addition, the content of polar materials in the studied oils was determined (Table 1). Almost all samples had a similar TPM content (4–5%) except the LO sample characterized by a high number of polar materials equal to 17.5 ± 0.5%. Usually, the increase in the content of polar materials is associated with oxidative processes occurring during heat treatment and frying of oils [22]. In this case, the high content of polar materials in LO seems to be due to the high content of polar nonfat substances (water, phospholipids, phenolic compounds) [23].

### 2.2. Oleogels Analysis

The microstructure of oleogels was studied to assess the effect of the type of oil medium on the morphology of the crystals. The morphology and size of crystals formed in oleogels affect the structural characteristics of the oleogels and, subsequently, the food products containing them [24,25]. The morphology of crystals of oleogels obtained with different oils was determined using polarized light microscopy.

**Figure 1:**
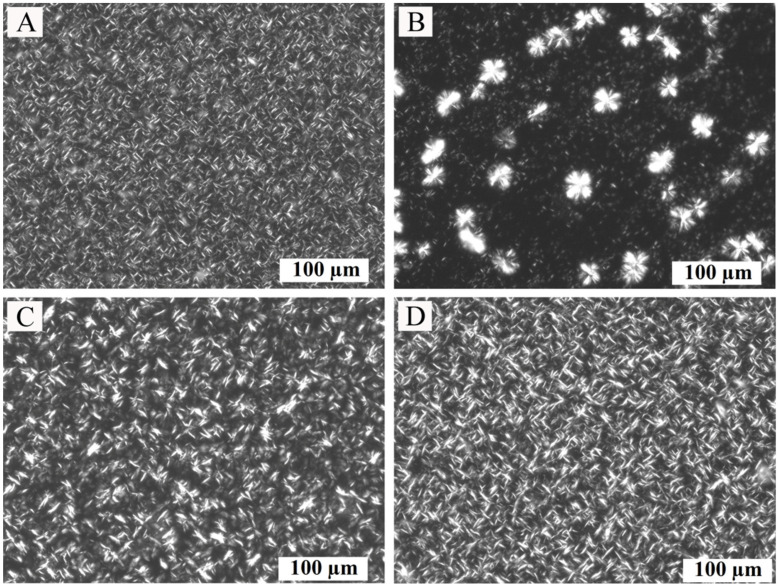
Microphotographs of oleogel samples: (**A**) FO-based oleogel, (**B**) LO-based oleogel, (**C**) SO-based oleogel, (**D**) OO-based oleogel. Scale bar: 100 µm.

As shown in Figure 1, the crystals have some differences in shape and size. The crystals formed in the oleogels based on FO, SO, and OO are similar to the needle-like shape, but according to [26], they are characterized by the platelet shape. The crystals of these oleogels have an identical appearance, in contrast to the crystals formed in LO oleogels. LO oleogels form several types of crystals, predominantly spherulitic in shape, which agrees with the study [24]. In FO, SO- and OO-based oleogels with a concentration of 6 wt.% of the structure form a compact oleogel mesh (Figure 1A,C,D); due to the high concentration of beeswax, a high degree of oversaturation is provided, which accelerates the nucleation and subsequent gelation. For a detailed analysis of the crystals formed in the investigated oleogels, their fractal dimension, lacunarity, and average length were calculated (Table 2).

Analysis of the fractal dimension by the box-counting method allows us to numerically estimate the uniformity of the solid mass distribution in the oleogels’ crystal network. The higher the fractal dimension, the more uniformly mass is distributed (or aggregated) in the network with fewer cavities [25]. In our case, the upper limit of D is 3 [25]. In addition to fractal dimension, the lacunarity (A) can also be used to describe certain properties of oleogels, as it provides detailed information on the distribution and homogeneity of cavities, in the crystal network, which, in turn, can be responsible for the macroscopic properties of the oleogels. Fractal dimension and lacunarity parameters had no significant differences (*p* > 0.05) among the FO- and OO-based samples. At the same time, the LO-based sample differed significantly from the other oleogels by these parameters, which suggests a more irregular structure with a large number of empty cavities, which, according to [25], may lead to low oil-binding capacity and the separation of oil from the oleogels with time, as well as to a decrease in hardness characteristics [27]. Another parameter characterizing the morphology of crystals, namely the distribution of crystals by size, is the average length (Lc). According to the results (Table 2), the smallest Lc values were observed for the FO oleogel crystals. The highest Lc values were observed for the LO-based oleogel. In the work [28], the authors found a relationship between the oil-binding capacity and Lc in oleogels structured with monoglycerides. Thus, the authors concluded that crystals with a lower Lc value exhibit better oil-binding capacity due to an increase in the surface area available for oil binding.

Studies were carried out to confirm the hypothesis that the distribution and crystal size of the structure-forming agent in oleogels were related to its oil-binding capacity and textural performance. Generally, oleogels contain a large amount of oil (on average, 90–96 wt.%), so they need a high oil-binding capacity for their successful use as an alternative to shortenings and spreads. The ability of oleogels to bind oil after centrifugation reflects their stability and shows the efficiency of structuring [29].

**Figure 2:**
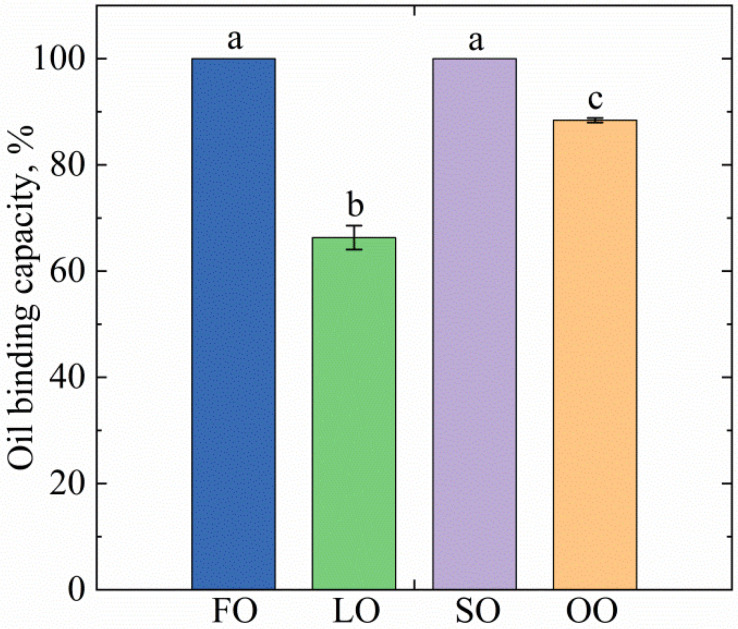
Oil-binding capacity of oleogels based on different oils: FO—fish oil, LO—linseed oil, SO—sunflower oil, OO—olive oil. The same letter indicates no significant difference (*p* < 0.01).

High OBC (Figure 2) was exhibited by the FO (100% ± 0.0%), SO (100% ± 0.0%), and OO (88.4% ± 2.5%)-based oleogel samples, which may be due to the more compact crystal network presented in Figure 1, which, as mentioned earlier, may influence the oil-binding capacity. According to [28], oil-binding capacity may correlate with the value of the fractal dimension, which is confirmed in our work, where R^2^ = 0.7988 for oil-binding capacity and fractal dimension and R^2^ = 0.8308 for oil-binding capacity and lacunarity. The low oil loss in oleogels based on vegetables [15,30] and animal oils [31] structured with beeswax is consistent with earlier studies, which, in particular, explains the differences in crystal shape and size in the oleogels. LO-based oleogels were found to have the lowest oil-binding capacity (less than 70%), which may be related to the spherulitic crystal shape, which, according to [27], is not favorable for the retention of large amounts of oil. However, this contradicts the study [24], where the linseed oil-based oleogels exhibited an OBC of around 90%. According to [32], when varying the ratio of individual components in the oleogels, the values of oil loss can undergo significant changes. Based on this, it can be assumed that the low OBC could be related to a certain ratio of fatty acids of linseed oil in the oleogel. Another possible cause of the low oil-binding capacity of linseed oleogels could be the presence of various impurities in the composition of the oils. To solve this problem, either oil without impurities should be used, or additional components in the composition of oleogels should be used. The lecithin added to oleogels can be one such component, which can change the morphology of the crystals and give additional crystal network strengthening, thereby reducing oil losses [29].

The texture of the studied oleogel samples was characterized using firmness, Young’s modulus (E), and Yield value (YV) indices.

**Figure 3:**
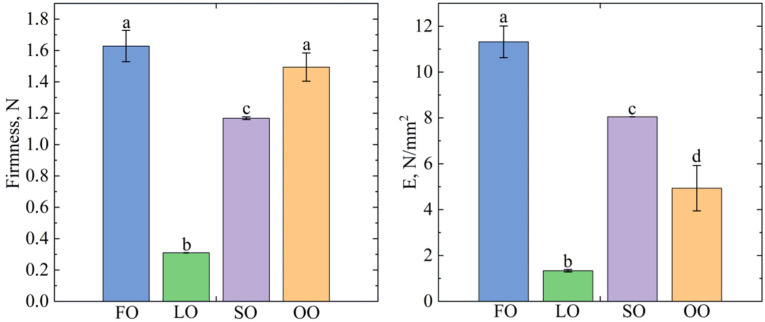
Texture properties of the oleogels produced with different oils: FO—fish oil, LO—linseed oil, SO—sunflower oil, OO—olive oil. The same letter indicates no significant difference (*p* < 0.01).

As the same structure-forming agent, beeswax, was used for all the samples, the differences in textural properties could be caused by the variety of fatty acid composition of the oils used as the dispersion medium. According to [33], if the hardness can be measured for all samples, it shows that they have solid-like properties. The results (Figure 3) showed that the hardness for the FO samples is (1.63 N ± 0.11 N), slightly lower for the OO (1.49 N ± 0.10 N) and SO samples (1.17 N ± 0.01 N), and lowest for the LO sample (0.31 N ± 0.01 N). According to [33], high hardness values are considered to be a desirable property of oleogel, as it allows the use of less structuring agents to achieve rheological characteristics similar to solid fats without significantly affecting the sensory characteristics of the product. Young’s modulus values differed slightly, with the highest value for oleogel FO (11.31 N/mm^2^ ± 0.69 N/mm^2^), similarly to hardness, and the lowest value for oleogel LO (1.33 N/mm^2^ ± 0.05 N/mm^2^). The high hardness values but low elastic modulus values of the OO sample indicate that the sample is similar in hardness to oleogel FO, but less elastic.

According to the classification described in [34] and the obtained YV values (Table 3), the texture of the three oleogel samples based on FO, SO, and OO can be classified as “satisfactory plastic and spreadable” and the LO oleogels as “very soft, not spreadable.” In particular, this characteristic can be useful when selecting oleogels to replace solid fat in food products, taking into account the individual characteristics of the end product. When analyzing the results of microscopy, where the average crystal length decreases in the series LO > SO > OO > FO, and hardness values, the oleogels with smaller crystals form stronger gels, which also confirms the conclusion put forward in [35] about the influence of crystal size on the textural properties of oleogels. In our opinion, this pattern may be related to the larger surface area in small crystals. Probably, certain fatty acids or polar components present in oils influence the morphological features of crystals, thereby changing the textural properties of oleogels. For example, work [36] indicates a significant influence of polar components on the structure of the oleogel network, which in turn leads to significant changes in the properties of oleogels, including textural properties. It is worth noting that, despite the differences in the hardness of oleogels, they can all be recommended as a substitute for solid fats, as shortenings and margarine with different textural properties can be used in the composition of food products.

**Figure 4:**
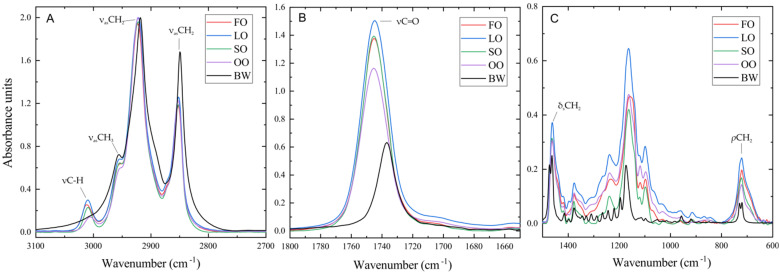
Averaged FTIR spectra of the oleogels and beeswax: (**A**) region IR spectrum 3100–2800 cm^−1^; (**B**) region IR spectrum 1800–1600 cm^−1^; (**C**) region IR spectrum 1500–600 cm^−1^.

FTIR spectroscopy was used to evaluate the effect of oil type on the crystallization of beeswax in oleogel (Figure 4A–C). Spectra of all samples of oils and oleogels based on them were obtained. Three regions can be distinguished in the IR spectrum of the oleogels: 3100–2800 cm^−1^ (Figure 4A), 1800–1600 cm^−1^ (Figure 4B), and 1500–600 cm^−1^ (Figure 4C). The first region includes vibrations of nonpolar aliphatic groups, the second region includes carbonyl group stretching absorption (1750 cm^−1^), and the third is a fingerprint region characterizing strain and skeletal vibrations of multi-atomic systems.

Statistically significant changes in the position of the absorption bands were revealed only for the vibrations of nonpolar aliphatic groups.

As shown in Figure 4, all the main peaks of beeswax are shifted toward lower wavenumbers. It shows the lower vibration energy of its functional groups compared to the oleogels. Figure 4A shows that the absorption band of asymmetric stretching vibrations of the CH_2_ group (ν_as_CH_2_) of beeswax is more intense than this absorption band of oleogels, indicating a greater length of the hydrocarbon chain of wax. Wax is also characterized by a less intensive peak in the carbonyl group stretching absorption region (νC = O), which indicates that possible changes in the position of this peak are mainly related to the changes in the characteristics of the carbonyl groups of triglycerides of oils.

**Figure 5:**
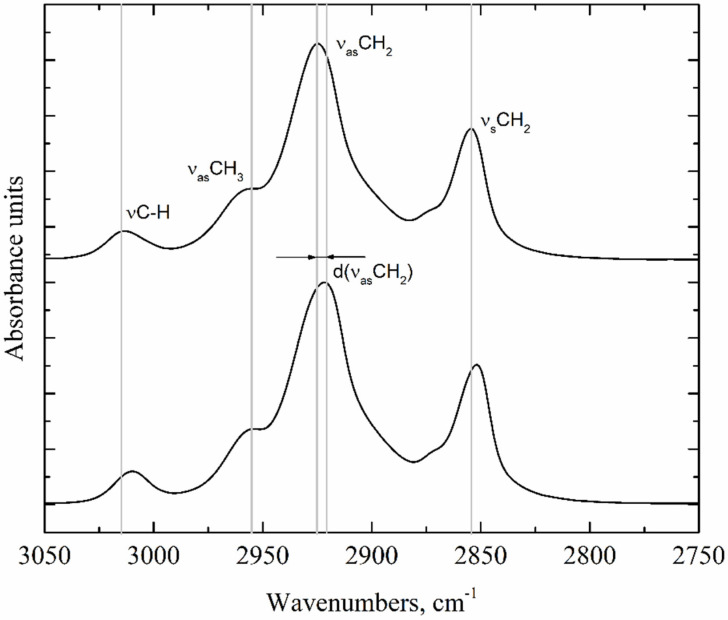
Illustration of the waveband difference calculation principle (νCH—CH stretching vibration of alkenes, ν_as_CH_3_—CH_3_ group asymmetric stretching vibration, ν_as_CH_2_—CH_2_ group asymmetric stretching vibration, ν_s_CH_2_—CH_2_ group symmetric stretching vibration, and d (ν_as_CH_2_)—intra-spectral difference.

The positions of the absorption bands of the main functional groups in the oil, as well as the shift (d) in these bands during gel formation, were studied (Figure 5).

Statistically significant shifts were found only for the C-H stretching vibration of alkenes (νC-H) absorption bands at 3005–3011 cm^−1^, CH_3_ asymmetric stretching vibration (ν_as_CH_3_) at 2953 cm^−1^, CH_2_ asymmetric stretching vibration (ν_as_CH_2_) at 2920–2925 cm^−1^, and CH_2_ symmetric stretching vibration (ν_s_CH_2_) at 2851–2854 cm^−1^ (Figure 5) [37]. The absence of significant changes in the absorption bands of polar groups indicates that their vibrations are not sensitive to any conformational change in the oleogelation by beeswax. The positions of the absorption bands for the studied samples are given in (Table 4) and correspond to the expected positions of the absorption bands for lipids.

The highest changes in the positions of the absorption bands are characteristic of the ν_as_CH_2_ and ν_s_CH_2_ vibrations. In all cases, these bands shift toward lower wavenumbers, which indicates a decrease in the vibrational energy of these bonds and the formation of more ordered structures of the hydrocarbon chain. The largest shift in these absorption bands is shown for the SO-based oleogel and is −4.672 cm^−1^ and −3.301 cm^−1^, respectively. The smallest shift is characteristic of FO-based oleogels and is −4.672 cm^−1^ and −3.301 cm^−1^, respectively. All samples are characterized by a greater shift in the asymmetric absorption band than the symmetric one. There are significantly smaller, but statistically significant, changes in the position of the νC-H and ν_as_CH_3_ absorption bands. The CH_2_ rocking vibration (*ρ*CH_2_, 722 cm^−1^) and CH_2_ and CH_3_ bending (δ_s_CH_2_, both at 1461 cm^−1^) peaks of all oleogel samples are not split, which is specific for hexagonal and triclinic but not orthorhombic packing [38]. In contrast, these peaks for the beeswax sample are separated, which indicates the orthorhombic shape of its crystals. The absence of orthorhombic crystals in oleogel samples is also confirmed by the results of polarized light microscopy (Figure 1) [39].

**Figure 6:**
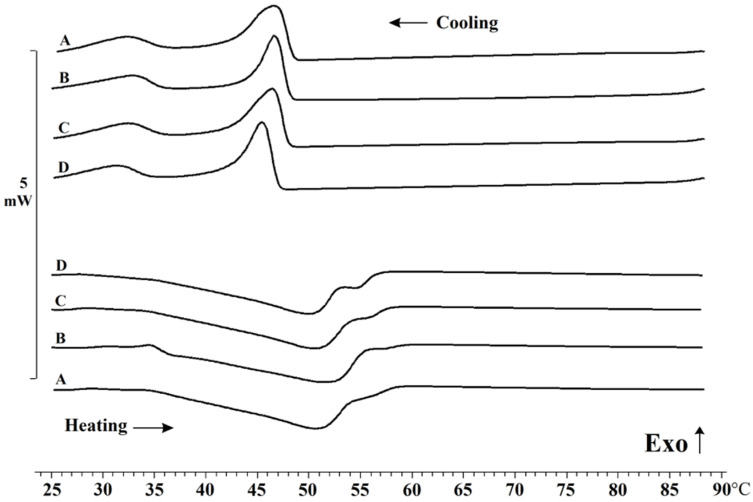
Heating and cooling thermograms of the oleogels (A—fish oil gel, B—linseed oil gel, C—sunflower oil gel, D—olive oil gel).

Melting and crystallization temperatures are important qualitative properties of fats, which determine the field of their application [30]. The thermal behavior of oleogels based on oils with different fatty acid compositions (Table 1) was characterized using DSC, as shown in Figure 6 and Table 5.

The onset temperature of crystallization varied as follows OO < SO < LO < FO. The melting temperature had insignificant differences OO < SO < FO < LO. In general, no significant differences in the position of the oleogel peaks were found, so we can conclude that there is no reliable influence of the fatty acid composition of the oils.

The absence of changes In melting and crystallization peaks between samples is consistent with the work [40], in which similar thermograms of oleogels, based on vegetable oils, were obtained, and it was shown that the thermal curve depends largely on the concentration and type of structure-forming agent, regardless of the oil phase. At the same time, our study found an additional melting peak around 37 °C in the thermogram of LO oleogels, which may be an indication of the formation of two types of crystals (Figure 1B). All of the oils used contained large amounts of unsaturated fatty acids, but the oleogels based on them had high melting temperatures, exceeding the melting temperatures of some animal fats, such as pork fat [41]. Due to their high melting temperatures, these oleogels must be used for certain product types in which fats of this type are required.

### 2.3. Correlational Analysis

**Figure 7:**
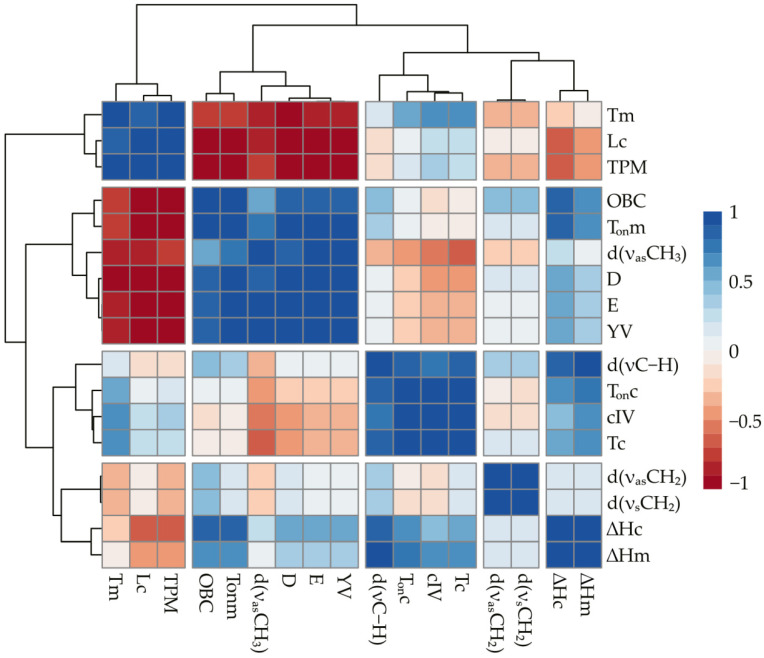
Correlation plot of the main parameters (positive correlations are shown in blue, negative correlations are shown in red).

Correlation analysis of the data was carried out to summarize the obtained data and identify common patterns for beeswax oleogels based on various edible oils, and a heatmap was constructed for all the obtained values. Figure 7 shows that the studied indicators can be divided into several groups depending on their correlation coefficient.

First, it should be noted that the values of the absorption band shifts for the ν_as_CH_2_ and ν_s_CH_2_ bonds are only in close correlation with each other. In this regard, we can conclude that the oil type in the beeswax oleogel is not related to changes in the vibrational characteristics of the hydrocarbon chain of the beeswax molecules during crystallization. The absence of changes in melting and crystallization peaks between samples is consistent with the work [40], which obtained similar thermograms of oleogels based on vegetable oils and showed that the thermal curve largely depends on the concentration and type of gelating agent, regardless of the oil phase. TPM, crystal size, and T_m_ can be distinguished into separate clusters with a close positive correlation with each other. Thus, an increase in oil polarity is associated with increases in melting temperature and crystal size in oleogels. In its turn, an increase in these values is connected with decreases in strength parameters (YV, E) and oil binding. The negative correlation between the crystal size of wax oleogel and its strength has been shown in various studies [42] and is an expected result. However, according to the literature, increasing the TPM value should increase the strength of the oleogel. A possible explanation for the discrepancy between our data and those previously obtained may be that the method for determining TPM is nonspecific. In the experiment [16], the content of polar material was increased by adding free fatty acids to the oil, which are effective gelators by themselves. In our study, the high values of polar materials could be related to both the nature of the oil [43] and to the presence of nonlipid impurities [23], which are not gelling agents and could potentially degrade the oleogel structure. The T_m_ value has a close positive correlation with cIV, that is, the degree of oil unsaturation. It is also shown that the T_c_ and T_on_c values closely correlate with cIV. The strength characteristics of beeswax-based oleogel are in close positive correlation with the oil-binding capacity. Significant correlations with these indices are shown for fractal dimension (D) and melting onset temperature (T_on_m). In addition, ΔHc is closely correlated with oil-binding capacity.

## 3. Conclusions

The study evaluated the effect of highly unsaturated oils in combination with beeswax, acting as a structure-forming agent, on the gelling properties of oleogels.

Based on the results of the fatty acid composition evaluation of the studied oils, the content of saturated, monounsaturated, and polyunsaturated fatty acids in the oils was determined, and the iodine value was calculated. The content of total polar materials was shown, and significant differences were revealed depending on the oil type. As a result of the conducted studies of oleogels, it was shown that the oil type does not affect the vibrational characteristics of the methyl groups of the hydrocarbon chain of substances in the composition of beeswax, and the formation of oleogel leads to similar changes in all oils.

Based on the microscopy analysis, differences in the shape and average length of crystals were revealed, and changes in fractal dimension and lacunarity were shown. The work showed the relationship between the average length of crystals, the total content of polar materials, and indicators of the mechanical strength of oleogels. The results of this work indicate that to obtain the hardest beeswax oleogels, it is necessary to use oils with the lowest content of polar materials and to provide conditions for the formation of the smallest crystals.

The textural properties of the FO-, OO-, and SO-based oleogels tested were characterized as “satisfactory plastic and spreadable” and the LO-based oleogel as “very soft, not spreadable.” The analysis of oil-binding capacity showed a high oil binding result for all samples except LO. The determination of the phase transition temperatures of the oleogels revealed similar melting peaks and crystallization among all the samples due to the added structure-forming agent. In the LO-based oleogel, an additional peak presumably related to the presence of several types of crystals was revealed.

The relationship between the iodine value and thermal properties of oleogels, particularly melting and crystallization temperatures, was also revealed. The obtained data can serve as a basis for targeted changes in the properties of food oleogels for different types of food products. Thus, for products requiring the use of oleogels with low mechanical characteristics, it is advisable to produce combinations of oils with a high content of polar materials, while ensuring a high melting temperature by reducing the iodine value. On the contrary, when the iodine value is high, the mechanical characteristics of oleogels can be increased by reducing the content of polar materials.

## 4. Materials and Methods

### 4.1. Materials

Beeswax (BW) was purchased from a local apiary (Dom voska, Russia) and had the following composition (% from identified substances): hydrocarbons (13.1%), esters (62%), free fatty acids (20.5%), alcohols (4.4%) [12]. Edible oils (sunflower oil—SO, olive oil—OO, linseed oil—LO, fish oil—FO) were purchased at a local supermarket (Moscow, Russia). Reagents used for chromatography: standard mixture FAME 37 Component mix in dichloromethane (Bellefone, PA, USA); undecanoic acid standard (C11-ME) (Bellefone, PA, USA); methanol, stabilized chloroform, acetyl chloride, and hexane were of analytical purity (Sigma-Aldrich, MO, USA).

### 4.2. Edible Oil Analysis Methods

#### 4.2.1. Fatty Acid Composition

The fatty acids composition was determined according to [44] using an Agilent Technologies 7683B Series gas chromatograph (USA) with a flame ionization detector and a 100 m Agilent J&W GC Columns Select FAME, 25 mm × 0.25 µm column (Netherlands). Oil samples (10–15 mg) were placed in a 5 mL tube, and then 2 mL of methanol, 20 µL of chloroform, and 2 µL of acetyl chloride were added. The tube was sealed tightly with a lid and spacer and placed in a thermostat (Binder FED 53, Germany) at 80 °C for 1 h. After cooling (about 10 min), 2.5 mL of hexane and 70 µL of distilled water were added to the samples. The samples were covered again and stirred on a vortex for about 30 s. The samples were allowed to stand for about 5 min, and then 1 mL of the upper phase was transferred to the vial for analysis. The sample introduction volume was 1 µL, the mode was 30:1 flow division, the carrier gas was nitrogen, and the flow rate was 0.9 mL/min. The injector temperature was 260 °C and the detector temperature was 240 °C. Separation conditions: initial temperature of 140 °C (isotherm for 5 min) and then increasing at a rate of 4 °C/min to 220 °C, isotherm 25 min. Data were collected and processed using Agilent ChemStation Rev.B.04.03 software. Ratios of LC methyl esters were calculated using the internal normalization method.

#### 4.2.2. Total Polar Materials (TPM)

The total polar materials content (%TPM) of oils was determined using a Testo^®^ 270 device (Testo Inc., Germany). Each test sample was preheated and measured at 40–50 °C, as the working range in the device was 40–200 °C, according to [16]. Each sample was analyzed in three replicates, and the mean value with standard deviation was calculated.

#### 4.2.3. Calculated Iodine Value

The calculated iodine value (cIV) was determined according to AOCS, Cd 1c-85 with additional factors for very long fatty acids according to [45].

### 4.3. Oleogel Preparation

**Figure 8:**
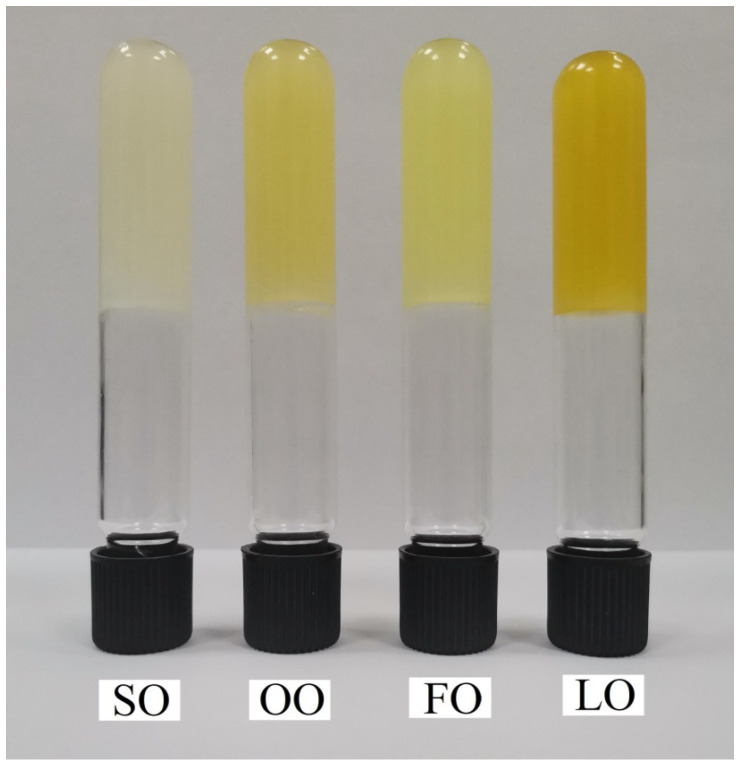
The appearance of oleogels produced with different oils: FO—fish oil; LO—linseed oil; CO—sunflower oil; OO—olive oil.

Oleogels were prepared according to the method described in [12]. The concentration of beeswax in all oleogels was 6% *w/w*. The heated oil with dissolved beeswax was poured into aliquots for each assay separately and cooled at a rate of 1 °C/min, followed by tempering at 23 ± 1 °C in a climatic chamber (Pol-Eko-Aparatura, Wodzisław Śląski, Poland) for 24 h. The appearance of the produced oleogels is shown on Figure 8.

### 4.4. Oleogel Analysis

#### 4.4.1. Microscopy

The microstructure of oleogels and crystal morphology was studied by polarized light microscopy (PLM) with a Zeiss Axio Imager.Z1 (Carl Zeiss Microimaging GmbH, Jena, Germany). Oleogel samples were melted to 90 °C, and then an aliquot was applied to a heated slide and covered with a coverslip. Samples were stored at 23 ± 1 °C for 24 h for crystallization. Microphotographs were taken with a Plan-Apochromat lens at 20× magnification. Each sample was analyzed in two replicates to determine crystal length (Lc), fractal dimension (D), and lacunarity (A) using ImageJ software (NIH, Bethesda, MA, USA) with the FracLac plugin. The “box-count” algorithm for determining the fractal dimensionality was used for the analysis. An additional dimension was added to the calculated value (D_b_) to represent the three-dimensional characteristics of the gel [46], as the determination performed by image analysis is based on two-dimensional space. The fractal dimension (D) was calculated using Equation (1).

(1) $$D=D_{b}+1$$

The details of D, A determination, and image preparation are described in [47,48].

#### 4.4.2. Oil binding Capacity (OBC)

The oil-binding capacity of oleogels was determined by centrifugation according to [15] with some modifications. The studies were performed by use of an SM-50SIA centrifuge (ELMI, Latvia). Approximately 1.0000 ± 0.0001 g of molten oleogel was placed in preweighed centrifuge tubes and weighed. The tubes were centrifuged at 11,000× *g* for 10 min. After centrifugation, the supernatant was decanted and the tubes with the remaining precipitate were resuspended. The oil-binding capacity was calculated according to [30]. The oleogel tubes were incubated for 24 h at 23 ± 1 °C before testing.

#### 4.4.3. Texture Analysis

The penetration test of a cone probe (60° cone, Perspex) using an EZ Test SX test machine (Shimadzu, Japan) was performed to determine the textural properties of oleogels prepared on oils with different contents of fatty acids, with beeswax as a texturing agent. Oleogels melted at 90 °C were poured into a reverse cone and cooled to 23 ± 1 °C in a Pol-Eko KK240 climatic chamber (Pol-Eko-Aparatura, Poland). Each sample was kept at this temperature for 24 h before analysis. The penetration rate was: 5 cm/min; test, 10 mm/min; distance, 9 mm. Hardness and Young’s modulus were measured automatically using Trapezium X software (Shimadzu, Kyoto, Japan). The yield value (YV, Nm^−2^ × 10^2^) was measured and characterized according to [49] with adaptations to automated testing machines [34].

#### 4.4.4. FTIR Spectroscopy

The samples of oils and oleogels were analyzed by FTIR spectroscopy using a Tensor 27 spectrometer (Bruker Optik GmbH, Billerica, MA, USA) and a MIRacleATR set-top box equipped with a germanium crystal (attenuated total reflectance method, ATR) using Opus 6.0 software. The measurements were performed similarly to the approach described in [50] with modifications. The oleogel samples were preheated to 90 °C and incubated for 30 min before the study until the crystals were completely melted. After that, 50–60 μL of the sample aliquot was quickly applied to the ATR crystal surface and incubated for 5 min until crystallization, and the spectrum was recorded. The studies were performed at 23 ± 1 °C. Each sample was analyzed in 4 repetitions. The obtained spectra were automatically processed, including the following steps: correction of ATR spectrum, correction of water vapor and carbon dioxide contribution from the air, baseline correction, and min-max normalization. The average value of the three samples closest to each spectra was used in the calculations.

#### 4.4.5. Differential Scanning Calorimetry (DSC)

The enthalpy (∆H), melting (T_m_), and crystallization (T_c_) temperatures were determined using a DSC 3 differential scanning calorimeter (Mettler-Toledo, Switzerland) equipped with a TC-45-MT intracooler (Huber, Germany) for cooling. Heat flux calibration with indium was performed before the study. Oleogel mass samples (6–8 mg) were weighed in 40 µL aluminum crucibles and sealed. The measurement cycle consisted of five stages: cooling, stabilization, heating, holding, and cooling. Heating and cooling were performed in the temperature range from 10 to 90 °C at a rate of 10 °C/min. The maximum heating temperature was 90 °C, which is related to the melting point of the used beeswax (71.2 °C) [12]. The obtained data were analyzed using STARe software (Mettler-Toledo). The average values of thermodynamic parameters measured in two repetitions are given as results.

### 4.5. Statistical Analysis

Statistical data analysis was performed using OriginPro 2018 software. For the correlation analysis, the data were pre-normalized. One-way analysis of variance (ANOVA) was used to assess the variation between groups followed by Fisher’s LSD post hoc test. The significance level was *p* < 0.05 with a 95% confidence level.

## Figures and Tables

**Table 1:** The chemical composition of the edible oils.

Fatty Acid Structure	OO	LO	FO	SO
**6:0**		-	-	-
**8:0**	-	-	0.02 ± 0.01	-
**10:0**	-	-	0.02 ± 0.01	-
**12:0**	-	-	0.04 ± 0.01	0.03 ± 0.00
**13:0**	-	-	-	-
**14:0**	0.03 ± 0.01	0.06 ± 0.01	3.85 ± 0.14	0.10 ± 0.02
**15:0**	-	0.03 ± 0.01	0.33 ± 0.11	-
**16:0**	12.56 ± 0.11	6.17 ± 0.14	9.66 ± 0.15	6.31 ± 0.31
**16:1**	0.15 ± 0.01	0.03 ± 0.01	0.37 ± 0.11	0.03 ± 0.01
**16:1 9-*c***	1.05 ± 0.12	0.12 ± 0.03	7.01 ± 0.21	0.12 ± 0.08
**17:0**	0.10 ± 0.02	0.09 ± 0.02	0.23 ± 0.05	0.05 ± 0.02
**17:1**	0.16 ± 0.04	0.05 ± 0.01	0.44 ± 0.05	0.04 ± 0.01
**18:0**	2.84 ± 0.10	4.32 ± 0.23	2.02 ± 0.10	3.54 ± 0.16
**18:1 9-*t***	0.21 ± 0.08	-	2.45 ± 0.20	-
**18:1 9-*c***	71.09 ± 0.47	20.76 ± 0.28	14.43 ± 0.27	27.31 ± 0.32
**18:1 11-*t***	2.28 ± 0.23	0.79 ± 0.13	4.26 ± 0.16	0.64 ± 0.07
**18:2 9-*c*, 12-*t***	0.04 ± 0.01	0.08 ± 0.02	0.06 ± 0.01	1.23 ± 0.14
**18:2 9-*t*, 12-*c***	-	-	0.03 ± 0.01	1.14 ± 0.08
**18:2**	8.08 ± 0.13	46.73 ± 0.32	1.83 ± 0.12	58.84 ± 0.48
**18:3 ω-3**	1.13 ± 0.21	20.43 ± 0.31	1.35 ± 0.17	0.29 ± 0.03
**20:1**	0.29 ± 0.07	0.34 ± 0.00	14.67 ± 0.21	0.30 ± 0.10
**20:5**	-	-	7.86 ± 0.19	-
**22:0**	-	-	0.04 ± 0.02	0.62 ± 0.11
**22:1**	-	-	10.91 ± 0.14	-
**22:4**	-	-	0.44 ± 0.05	-
**22:5**	-	-	1.51 ± 0.10	-
**22:6**	-	-	15.18 ± 0.24	-
**Total values**				
**SFA**	15.53	10.67	16.21	10.65
**UFA**	84.48	89.33	82.80	89.94
**MUFA**	75.23	22.06	54.54	28.44
**PUFA**	9.25	67.16	28.26	61.5
**cIV ^1^**	85.33	160.38	164.18	137.02
**TPM**	5.5 ± 0.5	17.5 ± 0.5	4.0 ± 0.5	5.0 ± 0.5

^1^ cIV—calculated iodine value, TPM—total polar matter, c—*cis* isomer, t—*trans* isomer; SFA—saturated fatty acids; UFA—unsaturated fatty acids; monounsaturated fatty acids; PUFA—polyunsaturated fatty acids; OO—olive oil; LO—linseed oil; FO—fish oil; SO—sunflower oil.

**Table 2:** Average length, fractal dimension (D), and lacunarity (A) of the oleogel microstructure.

Oil Type in Oleogel	Lc, µm	D	A
FO	7.1 ± 1.34 ^bcd^	2.82 ± 0.04 ^bc^	0.24 ± 0.05 ^bc^
LO	29.3 ± 4.01 ^acd^	2.49 ± 0.04 ^acd^	0.81 ± 0.05 ^acd^
SO	14.3 ± 1.74 ^abd^	2.75 ± 0.04 ^abd^	0.31 ± 0.05 ^abd^
OO	11.7 ± 1.72 ^abc^	2.81 ±0.04 ^bc^	0.24 ± 0.05 ^bc^

FO—fish oil, LO—linseed oil, SO—sunflower oil, OO—olive oil. Letters a, b, c, and d indicate samples with significant differences from each other (*p* < 0.05).

**Table 3:** Yield value for the oleogels and their classification.

Oil Type in Oleogel	YV (Nm^−2^ × 10^2^)	Assessment
FO	349.0 ± 2.3 ^bcd^	Satisfactory plastic and spreadable
LO	66.8 ± 7.3 ^acd^	Very soft, not spreadable
SO	250.5 ± 2.7 ^abd^	Satisfactory plastic and spreadable
OO	320.3 ± 1.5 ^abc^	Satisfactory plastic and spreadable

FO—fish oil, LO—linseed oil, SO—sunflower oil, OO—olive oil. Letters a, b, c, and d show statistical differences between samples (*p* < 0.01).

**Table 4:** Results of the FTIR spectra analysis of oil and corresponding oleogels.

Oil	State	Band Positions, cm^−1^			
		νC-H	ν_as_CH_3_	ν_as_CH_2_	ν_s_CH_2_
**Sunflower**	Oil	3008.938	2953.522	2924.939	2854.452
	Gel	3009.08	2953.589	2920.267	2851.151
	d(ν)	0.1423	0.0662	−4.672	−3.3008
**Fish**	Oil	3011.328	2953.151	2924.427	2853.931
	Gel	3011.465	2953.003	2922.849	2852.524
	d(ν)	0.1363	−0.1481	−1.5778	−1.4072
**Linseed**	Oil	3009.586	2953.671	2925.178	2854.447
	Gel	3009.637	2953.871	2923.337	2852.847
	d(ν)	0.0512	0.2003	−1.8411	−1.5997
**Olive**	Oil	3005.632	2953.605	2924.073	2853.96
	Gel	3005.573	2953.404	2921.91	2852.114
	d(ν) ^1^	−0.0588	−0.2015	−2.1633	−1.8465

d(ν)—difference between the band position in gel and in oil.

**Table 5:** Thermal parameters of oleogels produced with different oils: FO—fish oil; LO—linseed oil; SO—sunflower oil; OO—olive oil.

Sample	Heating			Cooling		
	T_on_	T_m_	∆Hm	T_on_	T_c_	∆Hc
FO	36.80 ± 0.4 ^bcd^	50.65 ± 0.6 ^bd^	−6.43 ± 0.5	48.48 ± 0.1 ^bcd^	46.52 ± 0.4 ^d^	4.76 ± 0.4 ^bd^
LO	34.84 ± 0.1 ^acd^	51.65 ± 0.9 ^acd^	−6.26 ± 0.6	48.15 ± 0.0 ^acd^	46.53 ± 0.0 ^d^	4.22 ± 0.2 ^a^
SO	36.19 ± 0.3 ^ab^	50.48 ± 0.3 ^b^	−6.36 ± 0.3	48.00 ± 0.1 ^abd^	46.52 ± 0.3 ^d^	4.58 ± 0.5
OO	36.04 ± 0.3 ^ab^	50.15 ± 0.2 ^ab^	−6.21 ± 0.2	47.02 ± 0.2 ^abc^	45.36 ± 0.1 ^abc^	4.24 ± 0.3 ^a^

(Temperature of melting—T_m_; enthalpy of melting—ΔHm; temperature of crystallization—T_c_; enthalpy of crystallization—ΔHc; temperature onset melting or crystallization—T_on_) of oleogels. Letters a, b, c, and d show statistical differences between samples (*p* < 0.05).

## References

[1] Pușcaș A., Mureșan V., Socaciu C., Muste S. (2020). Oleogels in food: A review of current and potential applications. Foods.

[2] Co E.D., Marangoni A.G. (2018). Oleogels: An Introduction. Edible Oleogels.

[3] Fayaz G., Calligaris S., Nicoli M.C. (2020). Comparative study on the ability of different oleogelators to structure sunflower oil. Food Biophys..

[4] Franco D., Martins A.J., López-Pedrouso M., Cerqueira M.A., Purriños L., Pastrana L.M., Vicente A.A., Zapata C., Lorenzo J.M. (2020). Evaluation of linseed oil oleogels to partially replace pork backfat in fermented sausages. J. Sci. Food Agric..

[5] Papadaki A., Kopsahelis N., Freire D.M., Mandala I., Koutinas A.A. (2020). Olive oil oleogel formulation using wax esters derived from soybean fatty acid distillate. Biomolecules.

[6] Zhang R., Zhang T., Hu M., Xue Y., Xue C. (2021). Effects of oleogels prepared with fish oil and beeswax on the gelation behaviors of protein recovered from Alaska Pollock. LWT.

[7] Kupiec M., Zbikowska A., Marciniak-Lukasiak K., Kowalska M. (2020). Rapeseed oil in new application: Assessment of structure of oleogels based on their physicochemical properties and microscopic observations. Agriculture.

[8] Li J., Yu H., Yang Y., Drummond C.J., Conn C.E. (2021). Effect of Crystallization State on the Gel Properties of Oleogels Based on β-sitosterol. Food Biophys..

[9] Hwang H.S., Gillman J.D., Winkler-Moser J.K., Kim S., Singh M., Byars J.A., Evangelista R.L. (2018). Properties of Oleogels Formed With High-Stearic Soybean Oils and Sunflower Wax. J. Am. Oil Chem. Soc..

[10] (1990). CODEX-STAN 210 Codex Standards for Fats and Oils from Vegetable Sources.

[11] Frolova Y.V., Kochetkova A.A., Sobolev R.V., Vorobyeva V.M., Kodentsova V.M. (2021). Oleogels as prospective nutritional ingredients of lipid nature. Vopr. Pitan..

[12] Sarkisyan V., Sobolev R., Frolova Y., Malinkin A., Makarenko M., Kochetkova A. (2021). Beeswax Fractions Used as Potential Oil Gelling Agents. J. Am. Oil Chem. Soc..

[13] Doan C.D., Tavernier I., Okuro P.K., Dewettinck K. (2018). Internal and External Factors Affecting the Crystallization, Gelation and Applicability of Wax-Based Oleogels in Food Industry. Innov. Food Sci. Emerg. Technol..

[14] Doan C.D., van de Walle D., Dewettinck K., Patel A.R. (2015). Evaluating the Oil-Gelling Properties of Natural Waxes in Rice Bran Oil: Rheological, Thermal, and Microstructural Study. J. Am. Oil Chem. Soc..

[15] Fayaz G., Goli S.A.H., Kadivar M., Valoppi F., Barba L., Calligaris S., Nicoli M.C. (2017). Potential Application of Pomegranate Seed Oil Oleogels Based on Monoglycerides, Beeswax and Propolis Wax as Partial Substitutes of Palm Oil in Functional Chocolate Spread. LWT.

[16] Gravelle A.J., Davidovich-Pinhas M., Zetzl A.K., Barbut S., Marangoni A.G. (2016). Influence of Solvent Quality on the Mechanical Strength of Ethylcellulose Oleogels. Carbohydr. Polym..

[17] Adili L., Roufegarinejad L., Tabibiazar M., Hamishehkar H., Alizadeh A. (2020). Development and characterization of reinforced ethyl cellulose based oleogel with adipic acid: Its application in cake and beef burger. LWT.

[18] Calligaris S., Mirolo G., da Pieve S., Arrighetti G., Nicoli M.C. (2014). Effect of Oil Type on Formation, Structure and Thermal Properties of γ-Oryzanol and β-Sitosterol-Based Organogels. Food Biophys..

[19] Xu Z., Liu S., Shen M., Xie J., Yang J. (2022). Evaluation of *trans* fatty acids, carbonyl compounds and bioactive minor components in commercial linseed oils. Food Chem..

[20] Dell’agli M., Bosisio E. (2002). Minor Polar Compounds of Olive Oil: Composition, Factors of Variability and Bioactivity. Stud. Nat. Prod. Chem..

[21] Giacintucci V., Di Mattia C.D., Sacchetti G., Flamminii F., Gravelle A.J., Baylis B., Dutcher J.R., Marangoni A.G., Pittia P. (2018). Ethylcellulose oleogels with extra virgin olive oil: The role of oil minor components on microstructure and mechanical strength. Food Hydrocoll..

[22] Bazina N., He J. (2018). Analysis of Fatty Acid Profiles of Free Fatty Acids Generated in Deep-Frying Process. J. Food Sci. Technol..

[23] Bekhit A.E.-D.A., Shavandi A., Jodjaja T., Birch J., Teh S., Ahmed I.A.M., Al-Juhaimi F.Y., Saeedi P., Bekhit A.A. (2018). Flaxseed: Composition, Detoxification, Utilization, and Opportunities. Biocatal. Agric. Biotechnol..

[24] Frolova Y., Sobolev R., Kochetkova A. (2021). Influence of Oil Combinations on the Structural Properties of Oleogels. E3S Web Conf. EDP Sci..

[25] Blake A.I., Co E.D., Marangoni A.G. (2014). Structure and Physical Properties of Plant Wax Crystal Networks and Their Relationship to Oil Binding Capacity. JAOCS J. Am. Oil Chem. Soc..

[26] Blake A.I., Marangoni A.G. (2015). Plant wax crystals display platelet-like morphology. Food Struct..

[27] Shi Z., Cao L., Kang S., Jiang S., Pang M. (2021). Influence of Wax Type on Characteristics of Oleogels from Camellia Oil and Medium Chain Triglycerides. Int. J. Food Sci. Technol..

[28] Palla C., de Vicente J., Carrín M.E., Gálvez Ruiz M.J. (2019). Effects of Cooling Temperature Profiles on the Monoglycerides Oleogel Properties: A Rheo-Microscopy Study. Food Res. Int..

[29] Okuro P.K., Tavernier I., bin Sintang M.D., Skirtach A.G., Vicente A.A., Dewettinck K., Cunha R.L. (2018). Synergistic Interactions between Lecithin and Fruit Wax in Oleogel Formation. Food Funct..

[30] Yılmaz E., Öğütcü M. (2014). Properties and Stability of Hazelnut Oil Organogels with Beeswax and Monoglyceride. JAOCS J. Am. Oil Chem. Soc..

[31] Öğütcü M., Arifoğlu N., Yilmaz E. (2015). Storage Stability of Cod Liver Oil Organogels Formed with Beeswax and Carnauba Wax. Int. J. Food Sci. Technol..

[32] Callau M., Sow-Kébé K., Nicolas-Morgantini L., Fameau A.L. (2020). Effect of the ratio between behenyl alcohol and behenic acid on the oleogel properties. J. Colloid Interface Sci..

[33] Choi K.O., Hwang H.S., Jeong S., Kim S., Lee S. (2020). The thermal, rheological, and structural characterization of grapeseed oil oleogels structured with binary blends of oleogelator. J. Food Sci..

[34] Do V.H., Mun S., Kim Y.-L., Rho S.-J., Park K.H., Kim Y.-R. (2016). Novel Formulation of Low-Fat Spread Using Rice Starch Modified by 4-α-Glucanotransferase. Food Chem..

[35] Yao Y., Zhou H., Liu W., Li C., Wang S. (2021). The Effect of Cooling Rate on the Microstructure and Macroscopic Properties of Rice Bran Wax Oleogels. J. Oleo Sci..

[36] Scharfe M., Ahmane Y., Seilert J., Keim J., Flöter E. (2019). On the Effect of Minor Oil Components on β-Sitosterol/γ-oryzanol Oleogels. Eur. J. Lipid Sci. Technol..

[37] George S. (2004). Infrared and Raman Characteristic Group Frequencies.

[38] Cameron D.G., Gudgin E.F., Mantsch H.H. (1981). Dependence of Acyl Chain Packing of Phospholipids on the Head Group and Acyl Chain Length. Biochemistry.

[39] Hondoh H., Ueno S. (2016). Polymorphism of Edible Fat Crystals. Prog. Cryst. Growth Charact. Mater..

[40] Pang M., Shi Z., Lei Z., Ge Y., Jiang S., Cao L. (2020). Structure and Thermal Properties of Beeswax-Based Oleogels with Different Types of Vegetable Oil. Grasas Y Aceites.

[41] Jiménez-Colmenero F., Cofrades S., Herrero A.M., Fernández-Martín F., Rodríguez-Salas L., Ruiz-Capillas C. (2012). Konjac gel fat analogue for use in meat products: Comparison with pork fats. Food Hydrocoll..

[42] Hwang H., Kim S., Singh M., Winkler-Moser J.K., Liu S.X. (2012). Organogel Formation of Soybean Oil with Waxes. J. Am. Oil Chem. Soc..

[43] Juárez M.D., Osawa C.C., Acuña M.E., Sammán N., Gonçalves L.A.G. (2011). Degradation in Soybean Oil, Sunflower Oil and Partially Hydrogenated Fats after Food Frying, Monitored by Conventional and Unconventional Methods. Food Control.

[44] Ayyildiz H.F., Topkafa M., Kara H., Sherazi S.T.H. (2015). Evaluation of Fatty Acid Composition, Tocols Profile, and Oxidative Stability of Some Fully Refined Edible Oils. Int. J. Food Prop..

[45] Ham B., Shelton R., Butler B., Thionville P. (1998). Calculating the Lodine Value for Marine Oils from Fatty Acid Profiles. J. Am. Oil Chem. Soc..

[46] Dàvila E., Parés D. (2007). Structure of Heat-Induced Plasma Protein Gels Studied by Fractal and Lacunarity Analysis. Food Hydrocoll..

[47] García-Armenta E., Picos-Corrales L.A., Gutiérrez-López G.F., Gutiérrez-Dorado R., Perales-Sánchez J.X.K., García-Pinilla S., Reynoso-García F., Martínez-Audelo J.M., Armenta-Manjarrez M.A. (2021). Preparation of Surfactant-Free Emulsions Using Amaranth Starch Modified by Reactive Extrusion. Colloids Surf. A Physicochem. Eng. Asp..

[48] Zhang Y., Dong M., Zhang X., Hu Y., Han M., Xu X., Zhou G. (2020). Effects of Inulin on the Gel Properties and Molecular Structure of Porcine Myosin: A Underlying Mechanisms Study. Food Hydrocoll..

[49] Haighton A.J. (1959). The measurement of the hardness of margarine and fats with cone penetrometers. J. Am. Oil Chem. Soc..

[50] Svečnjak L., Baranović G., Vinceković M., Prđun S., Bubalo D., Gajger I.T. (2015). An Approach for Routine Analytical Detection of Beeswax Adulteration Using FTIR-ATR Spectroscopy. J. Apic. Sci..

